# Predictors of Inguinal Lymph Node Metastasis in Penile Squamous Cell Carcinoma: Insights from a Single-Center Retrospective Study

**DOI:** 10.3390/jcm14092921

**Published:** 2025-04-23

**Authors:** Francesco Passaro, Luigi Napolitano, Antonio Tufano, Roberto La Rocca, Claudio Marino, Biagio Barone, Luigi De Luca, Ugo Amicuzi, Michelangelo Olivetta, Francesco Mastrangelo, Pasquale Reccia, Felice Crocetto, Lorenzo Romano, Francesco Paolo Calace, Lorenzo Spirito, Celeste Manfredi, Davide Arcaniolo, Antonio De Palma, Carmine Turco, Carmine Sciorio, Vincenzo Maria Altieri, Gennaro Mattiello, Ernesto di Mauro, Giuseppe Celentano, Sisto Perdonà

**Affiliations:** 1 Uro-Gynecological Department, IRCCS, Fondazione “G. Pascale”, 80131 Naples, Italyantonio.tufano91@gmail.com (A.T.); s.perdona@istitutotumori.na.it (S.P.); 2 Azienda Sanitaria Locale Salerno, ASL Salerno-DS66, Via Vernieri, 84124 Salerno, Italy; ernestodm9@gmail.com; 3 Department of Neurosciences, Reproductive Sciences and Odontostomatology, University of Naples Federico II, 80131 Naples, Italy; robertolarocca87@gmail.com (R.L.R.); marinoclaudio88@outlook.it (C.M.); u.amicuzi@gmail.com (U.A.); f.mastrangelo91@gmail.com (F.M.); felice.crocetto@gmail.com (F.C.); 4 Urology Unit, Department of Surgical Sciences, AORN Sant’Anna e San Sebastiano, 81100 Caserta, Italy; biagio.barone@aslnapoli1centro.it (B.B.); drmattiellogennaro@gmail.com (G.M.); 5 Department of Urology, A.O.R.N.A. Cardarelli, 80131 Naples, Italy; luigideluca86@gmail.com; 6 Urology Unit G. Fucito, Hospital and University Hospital, 84085 Salerno, Italy; olivetta.drmichelangelo@gmail.com; 7 Urology Unit, Department of Surgical Sciences, AORN dei Colli, Monaldi Hospital, 80131 Naples, Italy; reccia.pasquale1@gmail.com (P.R.); frap.calace@gmail.com (F.P.C.); 8 Urology Unit, Department of Woman, Child and General and Specialized Surgery, University of Campania “Luigi Vanvitelli”, 81100 Naples, Italy; loryromano@hotmail.it (L.R.); lorenzospirito@msn.com (L.S.); manfredi.celeste@gmail.com (C.M.); davide.arcaniolo@gmail.com (D.A.); 9 Department of Medical Oral and Biotechnological Science, Università degli Studi “G. d’Annunzio” of Chieti, 66100 Chieti, Italy; antonio.depalma.93@gmail.com; 10 Department of Urology, Ospedale del Mare, ASL NA1 Centro, 80147 Naples, Italy; car.turco87@gmail.com; 11 Urology Unit, A. Manzoni General Hospital, 23900 Lecco, Italy; carmine.sciorio@gmail.com; 12 Department of Medicine and Health Sciences “V. Tiberio”, University of Molise, 86100 Campobasso, Italy; vincenzomaria.altieri@gmail.com; 13 Urology Unit, Clinica Mediterranea, 80122 Naples, Italy; dr.giuseppecelentano@gmail.com

**Keywords:** penile cancer, lymph vascular invasion, squamous cell carcinoma

## Abstract

**Background:** Squamous cell carcinoma (SCC) of the penis accounts for approximately 95% of penile cancers and is associated with substantial morbidity and mortality. SCC typically develops in uncircumcised men, most commonly affecting the foreskin or glans. While slow-growing, early detection is crucial to improve survival outcomes. Risk factors include advanced age, lack of circumcision, poor hygiene, HPV infection (types 16 and 18), chronic inflammation, and smoking. **Methods:** We conducted a retrospective, single-center study at IRCCS Hospital “G. Pascale” of Naples, Italy, involving 59 patients treated between January 2015 and January 2023. The inclusion criteria were surgically treated primary tumors, confirmed SCC pathology, and pathologically verified inguinal lymph node metastasis (ILNM). We analyzed clinical variables including lymph node involvement, lymphovascular invasion (LVI), spongiosum corpus involvement (SCI), HPV infection, and tumor differentiation. Univariate and multivariate logistic regression analyses were performed to determine independent predictors of ILNM. **Results:** The mean age of patients was 66.67 ± 13.97 years. ILNM was confirmed in 24 patients (40.6%), while 35 (59.3%) had no lymph node involvement. Univariate analysis identified lymph node involvement at diagnosis (*p* = 0.005), LVI (*p* = 0.003), and SCI (*p* = 0.003) as significant predictors of ILNM. These factors were confirmed in the multivariate analysis, with lymph node involvement (*p* = 0.004), LVI (*p* = 0.025), and SCI (*p* = 0.028) as independent predictors. **Conclusions:** Lymph node status, LVI, and SCI are significant predictors of ILNM in penile SCC. Identifying these factors can aid in risk stratification, optimizing surgical decisions, and potentially reducing unnecessary morbidity. Further large-scale studies are recommended to validate these findings and refine prognostic models.

## 1. Introduction

Squamous cell carcinoma (SCC) of the penis represents about 95% of penile cancer, with an incidence of 1 per 100,000 males in Europe and the United States [1,2]. Several risk factors, including age (with a higher prevalence in men over 55), lack of circumcision, poor personal hygiene, human papillomavirus (HPV) infection, chronic inflammation, and smoking, have been identified [3]. This type of cancer typically progresses slowly, and early detection can significantly improve the prognosis, making early diagnosis critical for successful treatment outcomes. Despite its rarity, SCC is associated with high morbidity and mortality, largely due to its potential for lymphatic spread. The inguinal lymph nodes (ILN) are typically the first site of metastasis, and their involvement has a critical impact on prognosis and survival. The presence of metastasis in the ILN dramatically reduces survival rates, emphasizing the importance of accurate and early identification of nodal involvement [4]. ILN status is a relevant factor in clinical decision-making for penile cancer management and follow-up according to TNM classification. Although inguinal lymphadenectomy is associated with the best survival outcomes, it is also a procedure with significant potential complications, including skin necrosis, wound infection, lymphedema, seroma, lymphocele, and deep vein thrombosis [5]. These risks highlight the importance of carefully selecting patients for this procedure based on their risk profile and likelihood of lymph node metastasis [5,6,7]. Current research underscores that factors such as higher tumor stage, increased tumor grade, and the presence of vascular or lymphatic invasion are associated with an elevated risk of inguinal lymph node metastasis (ILNM) [8]. The aim of this retrospective study was to identify predictive factors for lymph node metastasis in penile SCC.

## 2. Materials and Methods

### 2.1. Patients

A total of 59 consecutive patients treated between January 2015 and January 2023 at IRCCS (Istituto di Ricovero e Cura a Carattere Scientifico) Hospital “G. Pascale” (Naples, Italy) were retrospectively enrolled. The inclusion criteria were (1) a surgically treated primary tumor, (2) histopathological confirmation of penile squamous cell carcinoma (SCC) by an expert uro-oncology pathologist, and (3) pathologically confirmed inguinal lymph node (ILN) metastasis via sentinel lymph node biopsy or prophylactic inguinal lymphadenectomy. The exclusion criteria included pelvic lymph node involvement or distant metastasis at diagnosis. The clinical and baseline characteristics—including age, hypertension, diabetes, phimosis, marital status, smoking history, HPV 16 infection, ILN involvement (confirmed by imaging or physical examination), TNM stage classification (8th edition), tumor differentiation, lymphovascular invasion (LVI), perineural infiltration (PNI), and surgical specimen size—were extracted from medical records and preoperative venous blood samples collected within 30 days prior to surgery. When multiple reports were available, data from the report closest to the surgery date were prioritized.

### 2.2. Ethical Aspects

This study adhered to the ethical principles of the Declaration of Helsinki. Written informed consent was obtained from all participants for data inclusion in a research database and subsequent scientific use. The institutional ethics committee approved the non-interventional, retrospective design (reference number 2308; 6 March 2023).

### 2.3. Study Outcome

The primary outcome was the identification of clinicopathological factors associated with ILN metastasis in penile SCC. The secondary outcomes included (1) the frequency of clinicopathological features (e.g., LVI, PNI) in penile SCC, (2) the association between surgical approach and postoperative outcomes, and (3) the diagnostic utility of imaging modalities in preoperative staging.

### 2.4. Study Design

This single-center retrospective study included patients managed surgically by a uro-oncology team. Surgical approaches encompassed penis-sparing techniques, partial amputation, or radical amputation with perineal urethrostomy. Sentinel lymph node biopsy or prophylactic inguinal lymphadenectomy was performed in accordance with the EAU guidelines for patients with stage ≥ T1G2 tumors and/or persistent lymphadenopathy post-antibiotic therapy. Histopathological confirmation of SCC was performed on formalin-fixed, paraffin-embedded tissue sections (4 μm thickness). Routine hematoxylin and eosin (H&E) staining was conducted using standardized protocols: tissues were deparaffinized, rehydrated, stained with Harris hematoxylin (5 min), differentiated in acid alcohol, counterstained with eosin (1 min), and mounted for light microscopy evaluation. For ambiguous cases or to refine diagnostic certainty, immunohistochemistry (IHC) was selectively employed using automated platforms (e.g., Ventana BenchMark Ultra). TNM staging followed the TNM stage classification 8th edition (2016), with pre-2016 cases reclassified accordingly.

### 2.5. Statistical Analysis

Descriptive statistics summarized continuous variables as mean ± standard deviation (SD) and categorical variables as frequencies (%). Normality was assessed using the Kolmogorov–Smirnov test. Continuous variables were assessed for normality using the Kolmogorov–Smirnov test. Normally distributed variables were compared using Student’s *t*-test, while non-normally distributed variables were analyzed with the Mann–Whitney U test. Categorical variables were evaluated via Chi-square tests. Univariate logistic regression identified individual risk factors for ILN metastasis; significant predictors (*p* < 0.05) were incorporated into a multivariate logistic regression model to control for confounders. Analyses were conducted in RStudio v.0.98 (R v.3.0.2) at a significance threshold of *p* < 0.05.

## 3. Results

**Figure 1:**
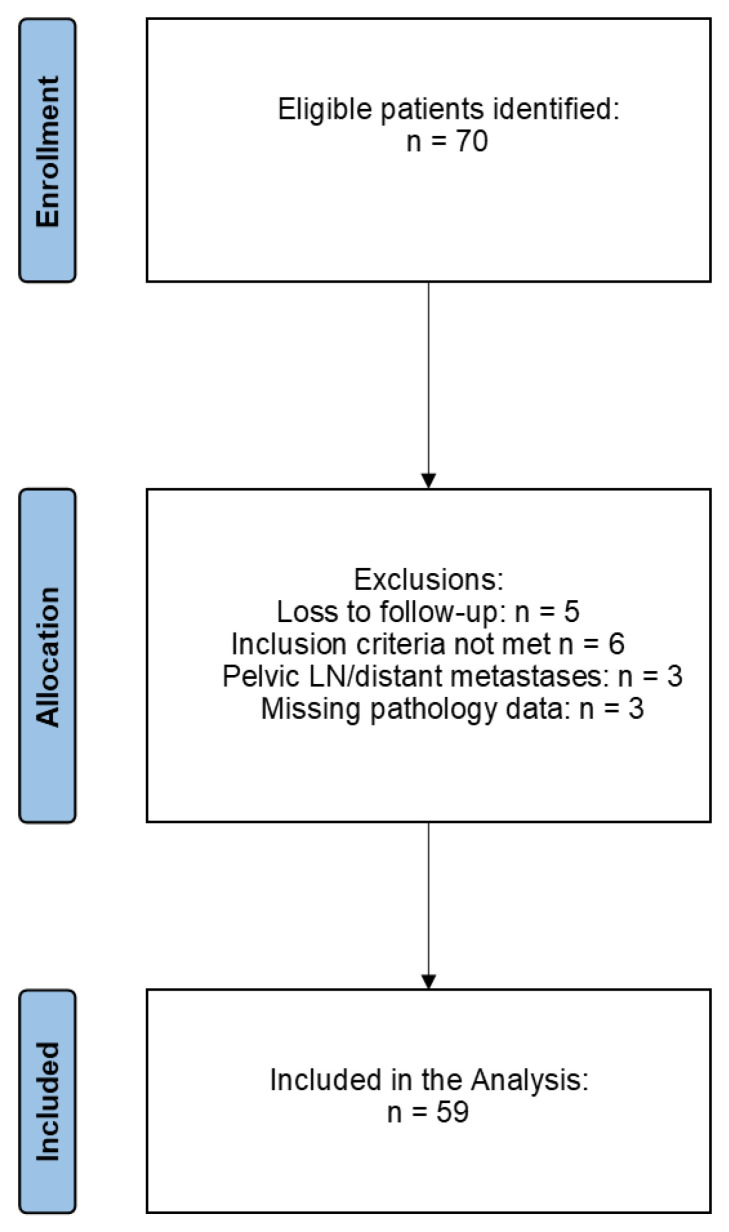
PRISMA flow diagram for patients included in the study.

Seventy patients were eligible for inclusion in this retrospective study. After screening, a total of 59 patients were included in this retrospective analysis (5 patients were lost to follow-up, and 6 patients did not meet inclusion criteria), with a mean age of 66.67 ± 13.97 years, reflecting the typical demographic profile for penile SCC (Figure 1) (Table 1). Among these patients, 24 (40.6%) were found to have inguinal lymph node (ILN) metastasis, as confirmed through sentinel lymph node biopsy or prophylactic inguinal lymphadenectomy, which are considered reliable methods for detecting metastasis. In contrast, the remaining 35 patients (59.3%) showed no evidence of lymph node metastasis, indicating that a substantial proportion of patients with penile SCC remain clinically node-negative. Univariate analysis was performed to explore potential correlations between various factors and ILN metastasis. This analysis highlighted three factors with statistically significant associations with ILN metastasis. The first of these was clinical lymph node involvement (cN), either on physical examination or through imaging tools, with a *p*-value of 0.005, indicating a strong correlation. Lymphovascular invasion (LVI) was the second factor, showing a *p*-value of 0.003, suggesting a critical role in metastasis development. Lastly, involvement of the spongiosum corpus was found to be significantly associated with ILN metastasis, also with a *p*-value of 0.003, underscoring its importance as a potential predictor. Given these significant findings in the univariate analysis, the three factors were subsequently included in a multivariate logistic regression model to determine their independent predictive power when controlling for confounding variables. The multivariate analysis confirmed all three as independent predictors of ILN metastasis. Specifically, the presence of lymph node involvement at diagnosis, assessed through objective examination or imaging techniques, remained significantly predictive of metastasis, with a *p*-value of 0.004. Lymphovascular invasion was also confirmed as an independent predictor, with a slightly reduced but still statistically significant *p*-value of 0.025. Finally, spongiosum corpus involvement was validated as a predictor, maintaining significance, with a *p*-value of 0.028 (Table 2). These results indicate that specific clinical and pathological characteristics—namely, lymph node involvement at diagnosis, lymphovascular invasion, and spongiosum corpus involvement—are strongly associated with inguinal lymph node metastasis in penile SCC. This analysis further supports their inclusion as essential factors for assessing metastatic risk, thus informing clinical decision-making regarding lymphadenectomy and surveillance strategies for patients with penile SCC.

## 4. Discussion

Penile cancer is a rare malignancy in Europe, with an incidence estimated at approximately 1 case per 100,000 men annually [3,9]. Although rare in developed countries, penile cancer accounts for a larger proportion of male cancers in developing nations, where it may represent up to 10% of all cancers in men [3]. The majority of penile cancers are squamous cell carcinomas (SCC), which constitute around 95% of cases [8,10].

The pathogenesis of penile SCC remains partially understood. It may arise de novo or evolve from penile intraepithelial neoplasia (PIN). High-risk human papillomavirus (HPV) infections, particularly strains 16 and 18, are strongly associated with SCC development [11]. The viral oncogenes E6 and E7 are key mediators in this process, disrupting the tumor suppressor genes p53 and RB1, which regulate cell proliferation [12]. The inactivation of these suppressor pathways results in uncontrolled cellular growth and the potential for malignant transformation [12].

Interestingly, HPV-DNA is detected in 22–72% of penile SCC cases, compared to 70–100% in PIN, suggesting that penile SCC may arise through both HPV-dependent and -independent mechanisms [11]. The prevalence of genital HPV-DNA in men across Europe varies, but most studies report an average prevalence of over 20%. Most HPV infections are transient and resolve spontaneously within 12 months, with a median duration of 5.9 to 7.5 months [11]. Factors such as older age and fewer sexual partners are associated with an increased likelihood of viral clearance. The quadrivalent HPV vaccine, which targets high-risk strains 6, 11, 16, and 18, has demonstrated a significant reduction in HPV-related genital diseases, including genital warts, with an efficacy of approximately 89.4% [13].

The widespread adoption of the HPV vaccine in men could potentially decrease the incidence of penile SCC and other HPV-associated lesions, such as genital warts and condylomas [11]. Penile SCC occurs predominantly in uncircumcised men, with neonatal circumcision recognized as a protective factor [3]. Key contributors to the increased risk in uncircumcised individuals include smegma accumulation and phimosis [3,14]. Smegma, a mixture of epithelial cell debris and bacterial byproducts, can cause chronic inflammation, potentially predisposing patients to both phimosis and malignancy [3,14]. Phimosis, the inability to retract the foreskin, is observed in 25–60% of patients with penile cancer and is often associated with increased infections and dysplastic changes in the preputial sac [2,3,13]. Furthermore, uncircumcised men are more susceptible to HPV infections compared to their circumcised counterparts.

Additional risk factors for penile SCC include smoking, HIV infection, poor genital hygiene, prior penile trauma, chronic balanitis, lichen sclerosus, and psoralen plus ultraviolet A (PUVA) therapy. Penile SCC typically presents between the ages of 50 and 70 years [2,3,8,9]. Lesions are most frequently located on the glans (48%), followed by the foreskin (21%), both glans and foreskin (15%), coronal sulcus (6%), and shaft (<2%) [3]. The clinical presentation is highly variable, ranging from small areas of erythema or induration to large, ulcerating, and infiltrative lesions. Symptoms such as itching, bleeding, foul-smelling discharge, and pain may develop as the disease progresses. Unfortunately, psychological factors can delay diagnosis, with an estimated 15–60% of patients postponing medical evaluation for over a year. Despite these delays, the majority of patients (66%) present with localized disease upon diagnosis [3].

The differential diagnosis for penile SCC includes premalignant and malignant lesions, infectious diseases, and inflammatory conditions. Non-invasive neoplastic conditions such as erythroplasia of Queyrat, Bowen’s disease, and Bowenoid papulosis should be considered. Verrucous SCC may resemble condyloma acuminata, while ulceration or secondary infections may mimic conditions such as syphilitic chancres or Haemophilus ducreyi chancroids. Inflammatory disorders such as psoriasis or lichen planus can also present with lesions that resemble SCC [3,15,16].

Penile SCC is histologically divided into several subtypes, including usual SCC (48–65%), basaloid carcinoma (4–10%), warty carcinoma (7–10%), verrucous carcinoma (3–8%), papillary carcinoma (5–15%), and mixed carcinomas (9–10%). Each subtype has unique histopathological characteristics, which are critical for determining tumor grade [13,17]. Grading is based on the degree of cellular differentiation, while staging is performed using the TNM classification system established by the American Joint Committee on Cancer. This system considers tumor invasion depth, as well as local and distant disease spread, to guide clinical management.

Lymph node involvement is one of the most critical prognostic factors in penile cancer. The 5-year survival rate drops to approximately 51% with the involvement of inguinal nodes, and survival prospects become markedly worse with pelvic node involvement [4,18,19,20]. Occult metastasis in clinically negative nodes has an incidence of around 10–20%, emphasizing the importance of the early detection of nodal metastasis [21,22]. Early lymphadenectomy has been shown to improve cancer-specific survival and may even be curative in patients with histologically positive nodes [21,23]. Despite attempts to identify inguinal node involvement using noninvasive methods, these techniques have proven ineffective, with clinical examinations historically showing a false positive rate of 50%. Consequently, current guidelines recommend invasive staging in cases with a clinically negative groin, based on primary tumor stage, grade, and lymphovascular invasion (LVI) status.

In our retrospective, single-center study, we identified predictive factors for lymph node metastasis in penile SCC. We initially evaluated ten variables, with only three showing statistical significance (*p* < 0.05) in the univariate analysis. These three variables—lymph node involvement at diagnosis (based on objective examination or imaging), spongiosum corpus involvement (SCI), and lymphovascular invasion (LVI)—were subsequently confirmed through multivariate regression analysis as independent predictors of lymph node metastasis.

These findings align with previous research on predictors of occult metastasis. For instance, an evaluation of the National Cancer Database by Winter et al. (2016) found LVI to be the strongest independent predictor of occult lymph node metastasis [24]. Similar conclusions have been drawn by other authors, including Ficarra et al. (2010) and Zekan et al. (2021), who have advocated for the inclusion of tumor grade and LVI in the European Association of Urology (EAU) risk stratification for clinically node-negative disease. However, the EAU guidelines present some limitations [25,26]. As noted by Graafland et al. (2010), 77% of patients classified as high-risk under the EAU criteria underwent negative bilateral lymphadenectomy, which resulted in increased morbidity due to unnecessary surgical intervention [27]. This emphasizes the ongoing need for additional predictive factors to enhance risk stratification and minimize the morbidity associated with overtreatment.

Tumor characteristics related to staging have also been assessed. Advanced tumor stage is often associated with specific structural involvement, such as spongiosum and cavernous corpus invasion. A recent systematic review by Hu et al. (2019) confirmed that corpus spongiosum invasion is a significant predictor of inguinal lymph node metastasis (OR 1.73, 95% CI 1.22–2.46; *p* = 0.002), a finding consistent with our results [28]. However, our study did not find cavernous corpus involvement to be a predictor of ILN metastasis, reflecting some variability across the literature [28].

The presence of nodal involvement remains one of the most critical factors influencing prognosis in penile SCC. Marconnet et al. (2010) and Ficarra et al. (2010) included nodal status in their nomograms for predicting patient prognosis [25,29]. However, subsequent studies have revealed conflicting results regarding its prognostic reliability, with authors like Alkatout et al. (2011) suggesting different outcomes [30].

This study has several limitations. First, its retrospective, single-center design introduces potential selection bias and limits the generalizability of our findings. Second, the small sample size (*n* = 59) reduces the study’s statistical power, particularly for detecting associations with rare variables (e.g., HPV 16+ status, urethral involvement). Third, HPV status was not systematically analyzed, despite its known role in penile SCC pathogenesis, which may confound the observed associations. Fourth, the histopathological data relied on institutional protocols without central pathology review, risking interobserver variability. Finally, the lack of long-term follow-up data precludes the assessment of survival outcomes or delayed metastasis.

## 5. Conclusions

Our study details the critical roles of lymphovascular invasion, corpus spongiosum involvement, and clinical nodal status in metastatic spread to regional lymph nodes into SCC. This is important to stratify patients to optimize their management. The inclusion of variables such as lymphovascular invasion and spongiosum involvement in risk assessment tools can refine prognostic models and provide a more robust framework for clinical practice. Our results also emphasize the need for a multidisciplinary approach to penile SCC, incorporating advanced pathological evaluation and tailored therapeutic strategies to maximize patient outcomes. Future research should aim to validate these findings in larger, multi-institutional cohorts and explore the potential role of emerging molecular markers in further enhancing prognostic accuracy.

## Figures and Tables

**Table 1:** Comparison of clinical and demographic characteristics between patients with pathological inguinal lymph node-negative (pILN−) and lymph node-positive (pILN+) penile squamous cell carcinoma. Data are presented as mean ± standard deviation (SD) for age, and as counts (percentages) for categorical variables.

		pILN−	pILN+	*p* Value
*n*		35	24	
*Age (mean(SD))*		69.91 ± 12.76	61.74 ± 14.57	0.028
*Surgery (%)*	Penile-sparing surgery	27 (77.1)	11 (45.8)	0.117
	Partial amputation	3 (8.6)	9 (37.5)	
	Radical amputation with perineal urethrostomy	5 (14.3)	4 (16.7)	
*Glans reconstruction (%)*		19 (54.3)	14 (58.3)	1.000
*Marital status (%)*	Unmarried	1 (2.9)	1 (4.2)	0.297
	Married	27 (77.1)	16 (66.7)	
	Widower	4 (11.4)	1 (4.2)	
	Unknown	3 (8.6)	6 (25)	
*Smoking (%)*	Never	7 (20)	5 (20.83)	0.237
	Current	16 (45.7)	14 (58.3)	
	Former	12 (34.3)	5 (20.8)	
*Phimosis (%)*		7 (20)	4 (16.7)	1.000
*Diabetes (%)*		4 (11.4)	6 (17.1)	0.312
*Hypertension (%)*		21 (60)	12 (50)	0.622

**Table 2:** Multivariate logistic regression analysis of factors associated with ILN metastasis in penile SCC. OR: odds ratio; CI: confidence interval; cN+: clinical nodal involvement; HPV 16+: human papillomavirus type 16 positive; LVI: lymphovascular invasion; PNI: perineural infiltration; T2/T3: tumor stage based on spongiosum/cavernous corpus involvement; G1/G2: well/moderately differentiated tumor; G3/G4: poorly differentiated/undifferentiated tumor.

Characteristic	OR	95% CI	*p*-Value
**cN+**			
**no**			
**yes**	6.56	1.87, 27.3	0.005
**Hpv 16+**			
**no**			
**yes**	1.69	0.56, 5.25	0.4
**Urethra involvement**			
**no**			
**yes**	4.03	0.78, 30.3	0.12
**Lymphovascular invasion**			
**no**			
**yes**	12.1	2.70, 86.8	0.003
**Perineural infiltration**			
**no**			
**yes**	5.44	1.11, 40.2	0.053
**Degree of tumor differentiation**			
**G1/G2**			
**G3/G4**	0.58	0.16, 1.94	0.4
**Cavernous corpus involvement (T3)**			
**no**			
**yes**	4.03	0.78, 30.3	0.12
**Spongiosum corpus involvement (T2)**			
**no**			
**yes**	6.29	1.97, 22.2	0.003
**Size of the operative piece**			
**<3 cm**			
**>3 cm**	0.8	0.21, 3.23	0.7
**Age (median = 68.5)**			
**>68.5**			
**<68.5**	1.54	0.54, 4.54	0.4
**cN+**			
**no**			
**yes**	38.7	4.67, 911	0.004
**Lymphovascular invasion**			
**no**			
**yes**	9.36	1.48, 85.1	0.025
**Spongiosum corpus involvement**			
**no**			
**yes**	6.17	1.30, 36.7	0.028

## Data Availability

The data presented in this study are available on request from the corresponding author.
